# Genome-Wide Association Analysis of Tolerance to Methylmercury Toxicity in *Drosophila* Implicates Myogenic and Neuromuscular Developmental Pathways

**DOI:** 10.1371/journal.pone.0110375

**Published:** 2014-10-31

**Authors:** Sara L. Montgomery, Daria Vorojeikina, Wen Huang, Trudy F. C. Mackay, Robert R. H. Anholt, Matthew D. Rand

**Affiliations:** 1 Department of Environmental Medicine, University of Rochester School of Medicine and Dentistry, Rochester, New York, United States of America; 2 Department of Biological Sciences, Genetics Program, and W. M. Keck Center for Behavioral Biology, North Carolina State University, Raleigh, North Carolina, United States of America; Duke University, United States of America

## Abstract

Methylmercury (MeHg) is a persistent environmental toxin present in seafood that can compromise the developing nervous system in humans. The effects of MeHg toxicity varies among individuals, despite similar levels of exposure, indicating that genetic differences contribute to MeHg susceptibility. To examine how genetic variation impacts MeHg tolerance, we assessed developmental tolerance to MeHg using the sequenced, inbred lines of the *Drosophila melanogaster* Genetic Reference Panel (DGRP). We found significant genetic variation in the effects of MeHg on development, measured by eclosion rate, giving a broad sense heritability of 0.86. To investigate the influence of dietary factors, we measured MeHg toxicity with caffeine supplementation in the DGRP lines. We found that caffeine counteracts the deleterious effects of MeHg in the majority of lines, and there is significant genetic variance in the magnitude of this effect, with a broad sense heritability of 0.80. We performed genome-wide association (GWA) analysis for both traits, and identified candidate genes that fall into several gene ontology categories, with enrichment for genes involved in muscle and neuromuscular development. Overexpression of glutamate-cysteine ligase, a MeHg protective enzyme, in a muscle-specific manner leads to a robust rescue of eclosion of flies reared on MeHg food. Conversely, mutations in *kirre*, a pivotal myogenic gene identified in our GWA analyses, modulate tolerance to MeHg during development in accordance with *kirre* expression levels. Finally, we observe disruptions of indirect flight muscle morphogenesis in MeHg-exposed pupae. Since the pathways for muscle development are evolutionarily conserved, it is likely that the effects of MeHg observed in *Drosophila* can be generalized across phyla, implicating muscle as an additional hitherto unrecognized target for MeHg toxicity. Furthermore, our observations that caffeine can ameliorate the toxic effects of MeHg show that nutritional factors and dietary manipulations may offer protection against the deleterious effects of MeHg exposure.

## Introduction

Methylmercury (MeHg) is a potent environmental neurotoxin that presents a risk to human health. MeHg exposures occur predominantly through dietary intake of fish species that harbor elevated levels of the organometal. Historic accidental MeHg poisonings in Minamata, Japan (1950's) and Iraq (1970's) demonstrated that the neurotoxic effects of MeHg result primarily from fetal exposures [1], [2]. In congenital Minamata disease, MeHg-exposed pregnant women with little to no neurological signs give birth to children with a range of severe clinical manifestations akin to cerebral palsy, including mental retardation, ataxia and motor deficits, growth retardation, speech and auditory deficits [3]. Clinical cases of Minamata disease, and limited samples of human fetal brain histopathology associated with them, have consolidated the notion that the developing nervous system is a target tissue for MeHg toxicity [4]–[6].

Large-scale epidemiologic studies that have investigated outcomes of prenatal MeHg exposure through seafood diets have yielded incongruent results with respect to neurological deficits [7], [8]. Subsequent studies have explored genetic predisposition and nutritional modifiers as factors that confer tolerance or susceptibility to MeHg among populations and in individuals [9]–[11]. In addition to neurotoxicity, recent population studies have identified MeHg effects on cardiovascular factors (e.g., heart rate variability and blood pressure) [12], [13] and the immune system [14], [15]. While less well studied, there is evidence that overall fetal and infant growth rates are inversely related to prenatal MeHg exposure [16], [17]. Given the prevailing focus on neural-specific mechanisms, the extent to which other developing organ systems are affected by MeHg during development has not been fully explored.

MeHg distributes rapidly and ubiquitously in living tissues and demonstrates an exceptionally high affinity for biological thiols, including glutathione (GSH) and protein thiols [18], [19]. As a result, the potential molecular and cellular pathways perturbed by MeHg during development are numerous. Thus, natural variation in phenotypic outcomes of MeHg exposure (*e.g.* tolerance or susceptibility) is the consequence of MeHg interaction with multiple gene products. However, few studies investigating MeHg mechanisms have focused on genetic variation as the underlying framework for variation in MeHg susceptibility. Conducting genome-wide association (GWA) analyses for MeHg susceptibility is challenging in human populations as both the extent of early developmental exposure and its postnatal manifestations are difficult to quantify, and environmental exposure in human populations cannot be controlled precisely. Thus, genome-wide studies on the genetic underpinnings of variation in MeHg exposure are best performed in a model system, where exposure conditions, environmental growth conditions and genetic background can be controlled and effects of MeHg exposure precisely quantified. Fundamental insights based on evolutionarily conserved biological processes can then be extrapolated to human populations.

*Drosophila melanogaster* presents an excellent genetic model system for quantitative genetic analyses of complex traits, and has resulted in the identification of genetic networks that underlie several stress responses, such as starvation resistance, chill coma recovery, startle behavior, oxidative stress sensitivity, and exposure to alcohol [20]–[23]. The recent establishment of the *Drosophila melanogaster* Genetic Reference Panel (DGRP), consisting of 205 wild-derived inbred fly strains with fully sequenced genomes, enables GWA studies in a population where all genetic variants are known [24], [25]. Linkage disequilibrium decays rapidly within *Drosophila* [24] and the limited population structure in the DGRP can be corrected for by taking into account segregating inversions and genomic relatedness [25]. In addition, the Drosophila model allows for rapid assessment of candidate genes through functional analyses of mutants.

Here, we have used the DGRP lines to perform a GWA analysis for variation in tolerance/susceptibility to MeHg toxicity during development. We measured the development of flies exposed to MeHg during larval and pupal stages by scoring eclosion (adult hatching) as a phenotypic endpoint. In addition, we examined development on MeHg food supplemented with caffeine, a previously identified dietary modifier of MeHg toxicity in fly development [26]. We find significant genetic variation in tolerance to MeHg as well as in modulation of MeHg toxicity by caffeine. Gene network and gene ontology analyses reveal, among others, an enrichment of genes related to development of muscle and the neuromuscular junction. We present a characterization of pupal MeHg phenotypes and functional analyses in mutant and transgenic flies that confirm a role for muscle development as a target for MeHg toxicity.

## Materials and Methods

### Drosophila Stocks

The following lines were obtained from the Bloomington *Drosophila* Stock Center (Indiana University): *Mi{MIC}kirre* ^MI07148^, (#41549), *Mi{MIC}kirre* ^MI00678^ (#41463) and *P{EP}kirre* ^G1566^ (#32593), *Mef2*-RFP (#26882), *y^1^w^67c23^* (#6599), *w^1118^*(#5905), *Mef2-Gal4* (#27390), and the entire *Drosophila melanogaster* Genetic Reference Panel (DGRP). The DGRP is a set of fully sequenced inbred lines generated by 20 generations of full-sib mating of progeny of wild-caught females from the Raleigh, NC population [24]. *UAS-GCLc5* and *UAS-GCLc 6* were kindly provided by Dr. William C. Orr, Southern Methodist University, Dallas TX [60]. All stocks were maintained at 25°C with 60% humidity and reared on cornmeal-molasses-agar culture medium.

### MeHg tolerance assays

Eclosion on MeHg food was assayed as previously described [27], [61]. Briefly, 30–50 first instar larvae (mixed sexes) were seeded on MeHg-containing media (0–15 µM). Assays for each treatment condition were performed on n = 150–300 larvae in three vials with 50 larvae each. On day 13 after larvae seeding, the number of eclosed adult flies were counted and expressed as percent of larvae applied to the food. To assess overall developmental tolerance on MeHg media, an eclosion index was calculated by normalizing the mean eclosion rate for each MeHg concentration to the eclosion on 0 µM MeHg for each strain. An overall eclosion index was then generated by summing the normalized percent eclosion values obtained on the 5, 10, and 15 µM MeHg treatments for each strain. For example, if a strain exhibits 95% eclosion on 5 µM MeHg, 50% eclosion on 10 µM MeHg and 5% eclosion on 15 µM MeHg, the eclosion index would be 95+50+5 = 150. Some DGRP strains demonstrated less than 60% eclosion on 0 µM MeHg (9 lines total), exhibiting poor baseline eclosion behavior. These lines were omitted from the analyses, for a total of 167 DGRP lines.

Parallel assays assessing the modulating effects of caffeine on eclosion rates were also determined on 0 µM and 10 µM MeHg with and without 2 mM caffeine (LKT Laboratories; St. Paul MN). This concentration of caffeine was previously characterized as a sub-toxic and tolerance-promoting dose in two wild-type *Drosophila* strains [26]. Eclosion values were normalized to the 0 µM MeHg condition for each strain. A caffeine difference index was determined for each line by subtracting the normalized eclosion rate on 10 µM MeHg alone from that on 10 µM MeHg+2 mM caffeine. Lines exhibiting 0% eclosion on 10 µM MeHg and 10 µM+2 mM caffeine were omitted from GWA analysis, leaving 139 lines for GWA analysis.

### Quantitative genetic analyses

We performed a mixed effects model analysis of variance (ANOVA) using the model *Y* = *µ*+*D*+*L*+*D×L*+*ε* to partition phenotypic variation, where *Y* is the response variable, *µ* is the overall mean, *D* is the fixed effect for dose, and *L* and *D×L* are the random effects for line and dose by line interaction. Significance of the effects was tested using type III *F* tests implemented in SAS Proc Mixed (SAS Institute). Broad sense heritability was calculated as 
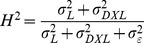
. For the effect of caffeine, because there was only a single dose of MeHg, a simpler model was fitted as *Y* = *µ*+*L*+*ε*, and 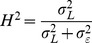.

### Genome-wide association analysis

We performed genome-wide association analysis on line means of the phenotype using the DGRP analysis portal (http://dgrp2.gnets.ncsu.edu; [24], [25]). Briefly, the hatching index or caffeine index was used to fit a mixed model for each variant in the form of *Y* = *µ*+*M+g*+*ε*, where *Y* is the line means adjusted for *Wolbachia* infection and five major inversion polymorphisms (*In(2L)t*, *In(2R)NS*, *In(3R)Y*, *In(3R)P*, *In(3R)Mo*) in the DGRP, *µ* is the overall population mean, *M* is the effect of DNA variant being tested, and *g* is a polygenic component with covariance between lines determined by their genomic relationship [25]. For Wolbachia infection adjustment we fit a linear model with the infection status and major inversion genotypes as covariates and the raw phenotypes as the response variable. Residuals from this linear model were used as inputs for GWAS. We performed 2,180,555 tests for association for the MeHg treatment alone, restricting the analyses to variants for which at least 6 lines contain the minor allele (for a minor allele frequency, MAF, >3.6%). We performed 2,357,353 tests for association for the MeHg+caffeine treatment, restricting the analysis to variants for which at least 4 lines contained the minor allele (MAF>2.9%).

### GeneMANIA network analysis

Polymorphism-based single marker analysis for MeHg and MeHg+caffeine was used to identify top candidate genes found to be associated with developmental tolerance to MeHg. Candidate genes were uploaded to the GeneMANIA prediction server (www.genemania.org), a web interface to identify networks of gene functions associated with a query list of genes [39]. Functional networks were derived using automatic query-dependent gene weighting and biological function-based gene ontology (GO) weighting to identify interactions based on co-expression, co-localization, genetic, and physical interactions of query and non-query genes related to biological integration networks. Outputs from GeneMANIA were constructed in tabular form and in graphical form using a Cytoscape plugin v3.1.0 (Cytoscape). Functional enrichment is based on the GO categories and is reported as Q-values of a false discovery rate (FDR) using a corrected hypergeometric test for enrichment. Coverage ratios for the number of annotated genes in the displayed network versus the number of genes with that annotation in the genome are also reported. Q-values are derived using the Benjamini-Hochberg procedure. Categories are displayed up to a Q-value cutoff of 0.1.

### Muscle phenotype characterization

*Mef2*>RFP L1 larvae were seeded onto 0, 10, and 15 µM MeHg media and monitored until pupae formation at 25°C. Stage 6–12 pupae were selected based on established developmental landmarks such as the appearance of green in Malpighian tubules, body color, eye color, bristles development and wing color [62]. Pupae were dissected from their case and positioned on a Superfrost microscope slide (VWR International; Radnor, PA) for fluorescent reporter imaging of the indirect flight muscles (IFMs) within the thorax. Phosphate-buffered saline (PBS) was added drop-wise to each dissected pupae to avoid desiccation. Fluorescent microscopy was performed with a Leitz Orthoplan 2 microscope (Leica Microsystems; Buffalo Grove, IL) equipped with a SPOT Insight QE 4.2 Camera (SPOT Imaging Solutions; Sterling Heights, MI) and imaging software using a 4× objective. Images were assembled in Microsoft PowerPoint.

### Functional validation of candidate genes

Functional validation of candidate genes related to muscle development was performed with eclosion assays using *kirre* ^MI07148^, *kirre* ^MI00678^ and *kirre* ^G1566^ mutants (Bloomington *Drosophila* Stock Center, Indiana University). Eclosion on MeHg food (0–15 µM) was assayed for the *kirre* mutants and the corresponding *y^1^w^67c23^* control strain. Experiments overexpressing *UAS-GCLc5* and *UAS-GCLc6* were conducted by using the muscle-specific *Mef2-Gal4* driver. Female *Mef2-GAL4* flies were bred with male *UAS-GCLc5*, U*AS-GCLc6* or *w^1118^* control flies, and progeny assayed for eclosion on MeHg media (0–25 µM). Rates of eclosion for indicated strains at each MeHg concentration are expressed in proportions (% eclosion). Assays were performed to achieve n = 150 larvae. Statistical consideration of differences between experimental and control fly strains are therefore comparisons of proportion values and not of continuous values. Since error determinations in proportion values become restricted at the edges (*i.e*. near 100%), an analysis of variance (ANOVA) was not used. Statistical analyses of eclosion assays were therefore done using a pairwise 2-tailed *z*-test, treating each MeHg concentration categorically. *p*-values of less than 0.01 were considered significant.

### Gene expression

*GCLc* and *kirre* gene expression was measured by quantitative real-time PCR (qRT-PCR) of RNA extracts isolated from first instar larvae or staged pupae. For the indicated genotypes, RNA was extracted by pooling 15–20 larvae or 20–25 pupae. The tissue was homogenized and RNA extracted with Trizol (Invitrogen; Grand Island, NY). qRT-PCR quantification was performed on a Bio-Rad CFZ Connect Real-Time PCR Detection System using CFX Manager software. cDNA synthesis and reverse transcription was performed in a Bio-Rad iScript SYBR Green one-step reaction (Bio-Rad; Hercules, CA). Twenty-five µL reactions containing 40 ng RNA template, iScript SYBR Green reaction mix (2x), iScript reverse transcriptase for one-step RT-PCR, and forward and reverse primers (10 µM final concentration) were used. The *Drosophila Rp49* gene was used for normalization of expression. Gene expression levels were determined by the comparative C_T_ method [63]. The following primers were used; *Rp49*: 5′-AGTATCTGATGCCCAACATCG-3′ and 5′-TTCCGACCAGGTTACAAGAAC-3′, *GCLc*: 5′-ATGACGAGGAGAATGAGCTG-3′, and 5′-CCATGGACTGCAAAATAGCTG-3′, *kirre*: 5′-TGGACTGGCCATTAATCTTACC-3′ and 5′-AACGATCGCCACCGAAAT-3′.

## Results

### Quantitative genetic analysis of natural variation in tolerance to MeHg during development

**Figure 1:**
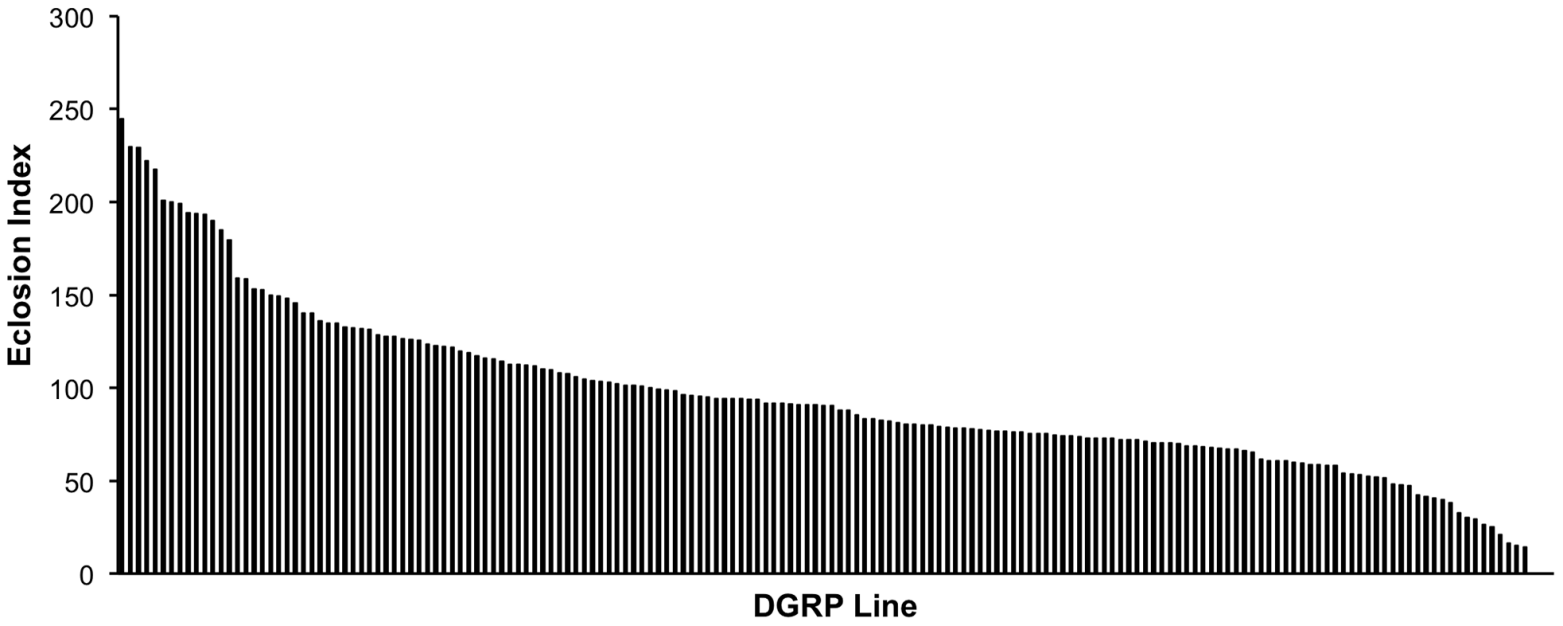
Genetic variation in MeHg tolerance during development. 176 DGRP lines were assayed in triplicate for eclosion on media containing 0, 5, 10 and 15 µM MeHg. A cumulative index (Eclosion Index) was generated by summing the percent eclosion on 5, 10 and 15 µM MeHg food for each strain (see methods). The histogram represents a rank ordering of the eclosion index for each of the DGRP lines.

**Figure 2:**
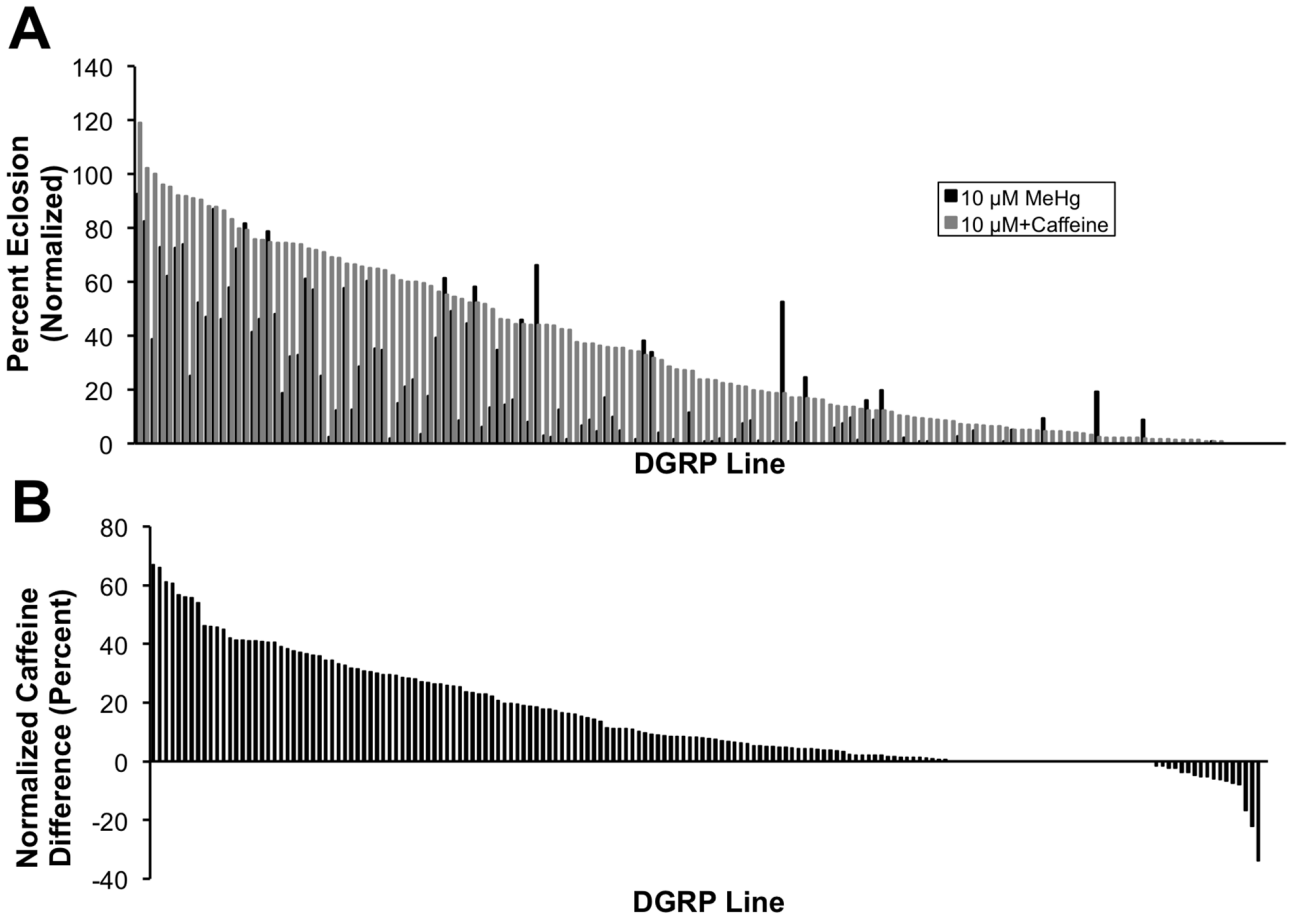
Caffeine effect on MeHg tolerance exhibits genetic variation. (A) Eclosion rates of DGRP lines were determined on 0 µM and 10 µM MeHg food with and without addition of 2 mM caffeine. The histogram is a rank ordered expression of eclosion rates on the MeHg+caffeine condition (gray bars) paired with the respective line on MeHg alone (black bars). (B) A caffeine difference index was determined for each DGRP line by subtracting the normalized eclosion rate on 10 µM MeHg from that on 10 µM MeHg+2 mM caffeine. Positive values indicate a beneficial effect of caffeine and negative values indicate a detrimental effect of caffeine relative to 10 µM MeHg alone. Lines showing 0% eclosion on both MeHg alone and MeHg+caffeine were omitted from the analyses leaving 139 lines for GWA analyses.

To characterize natural genetic variation in tolerance and susceptibility to MeHg during development, we examined 176 DGRP lines in an eclosion assay on four concentrations ([MeHg] = 0, 5, 10, 15 µM, Table S3) of MeHg-containing food. We found substantial phenotypic variation in susceptibility to MeHg as measured by an eclosion index (see Materials and Methods for definition) across the DGRP lines, ranging from 0 to 244.6 (Fig.1). ANOVA for variation in MeHg tolerance across the four concentrations of MeHg showed significant variation for Line and the Dose × Line interaction term (Table 1). The broad sense heritability (*H* ^2^) was 0.86, indicating a strong genetic component to variation in MeHg tolerance, which provides a basis for GWA analysis (Table 1). To explore dietary modulation of MeHg toxicity, we examined the effects of caffeine co-administration (Fig. 2A, B, Table S3), which has previously been shown to attenuate MeHg developmental toxicity in a limited set of fly lines [26], [27]. Addition of 2 mM caffeine to 10 µM MeHg supplemented food resulted in increased MeHg tolerance in the majority of the lines (Figure 2A, B), with only 12 lines exhibiting decreased MeHg tolerance with caffeine supplementation (Fig 2B). The variation in the modulating effect of caffeine can be seen clearly by subtraction of the eclosion rate on MeHg alone from that on MeHg+caffeine (Figure 2B). The modulating effect of caffeine on MeHg exposure varies significantly across the lines with *H* ^2^ = 0.80 (Table 2).

**Table 1:** ANOVA table for MeHg tolerance.

Source of Variation	Df	MS (Type III)	F	P value	σ^2^ (SE)
**Line**	172	0.0909	12.42	<0.0001	0.0299 (0.0035)
**Error**	346	0.0073			0.0073 (0.0006)

Phenotype  =  hatching rate for each replicate at 5, 10, 15 µM MeHg normalized to the line means of hatching rate at 0 µM MeHg. Residuals are weighted by the square of number of flies assayed.

**Table 2:** ANOVA table for caffeine effect.

Source of Variation	Df	MS (Type III)	F	P value	σ^2^ (SE)
**Dose**	2	66.4455	10788	<0.0001	Fixed
**Line**	172	0.1983	32.21	<0.0001	0.0155 (0.0027)
**Dose × Line**	344	0.0711	11.55	<0.0001	0.0231 (0.0019)
**Error**	3	0.0062			0.0062 (0.0003)

Phenotype  =  (hatching rate for each replicate at 10 µM MeHg + Caffeine normalized to the line means of hatching rate at 0 µM MeHg) – (line means of hatching rate at 10 µM MeHg normalized to the line means of hatching rate at 0 µM MeHg). Residuals are weighted by the square of number of flies assayed.

### Polymorphic markers associated with MeHg tolerance

We performed single marker GWA analyses to identify polymorphic markers (single nucleotide polymorphisms (SNPs), insertions and deletions) associated with MeHg tolerance and the modulatory effect of caffeine on MeHg toxicity. Line means for both traits were approximately normally distributed (Fig. S1); therefore, we did not transform the data. Quantile-quantile plots show a clear enrichment of variants with *p*-values <10^−4^ for MeHg tolerance, but no evidence for enrichment for the modulatory effect of caffeine (Fig. S2). In the MeHg tolerance GWA, one SNP (in *pHCl*) nearly met a Bonferroni-adjusted significance level, and 14 SNPs (in *pHCl*, *eco*, *CG44245* *CG33981* and *CG15221*) had false discovery rates (FDR) of FDR <0.2 (Table S1).

**Figure 3:**
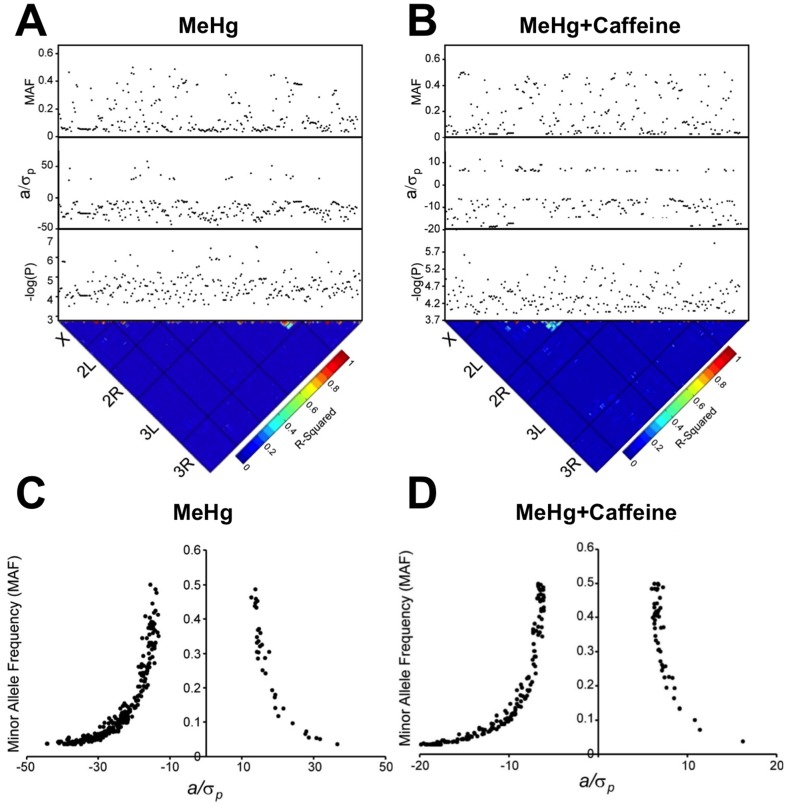
Genome-wide association analysis of eclosion on MeHg with and without caffeine supplementation. Single marker analyses using ANOVA of 2,180,555 (MeHg alone) and 2,357,353 (MeHg+caffeine) polymorphic alleles across 167 (MeHg alone) and 139 (MeHg+caffeine) DGRP lines, respectively, resolved (A) 350 and (B) 239 polymorphic markers (*p*<10^−4^, MAF>3%). Depicted is a heat map for linkage disequilibrium (LD) based on *r^2^* values where the black bars represent the five major *Drosophila* chromosome arms. Red indicates high LD, while blue indicates low LD. The black dots represent polymorphic marker associations for eclosion on MeHg or MeHg+caffeine. *p* values (log_10_(*p*)), effect size (*a*/*σ_P_*), and the minor allele frequency (MAF) are shown. (C,D) MAF *vs.* effect size. All 350 (C) or 239 (D) polymorphic markers associated with phenotypic variation for eclosion on MeHg or MeHg+caffeine, respectively, are depicted. *a*/*σ_p_* indicates effect size ([mean of major allele class – mean of minor allele class]/2), where negative *a*/*σ_p_* indicates the minor allele is associated with increased MeHg tolerance with respect to eclosion.

In *Drosophila*, we can use GWA analysis as an exploratory hypothesis-generating tool, and test hypotheses more rigorously in secondary screens using mutations or targeting RNAI for candidate genes implicated by the GWA analysis. Therefore, we used a lenient reporting threshold of *p*<10^−4^ for both GWA analyses (Tables S1, S2). We identified 350 polymorphisms in or near 145 genes associated with variation in eclosion rates on MeHg food (Fig. 3A) and 239 polymorphisms in or near 106 genes (Fig. 3B) associated with the modulatory effect of caffeine on MeHg treatment. Most polymorphisms associated with the two analyses had MAF <0.15, and, as expected, we found inverse relationships of effect size and allele frequency (Figs. 3C, 3D). Rarer alleles were associated with greater tolerance to MeHg and modulation of the effect by caffeine with respect to larva-adult viability for a majority of the polymorphisms (Figs. 3C, 3D), suggesting these alleles have other deleterious effects on fitness. There was little linkage disequilibrium between the most significant polymorphisms (Figs. 3A, 3B).

### Candidate genes associated with MeHg tolerance

**Figure 4:**
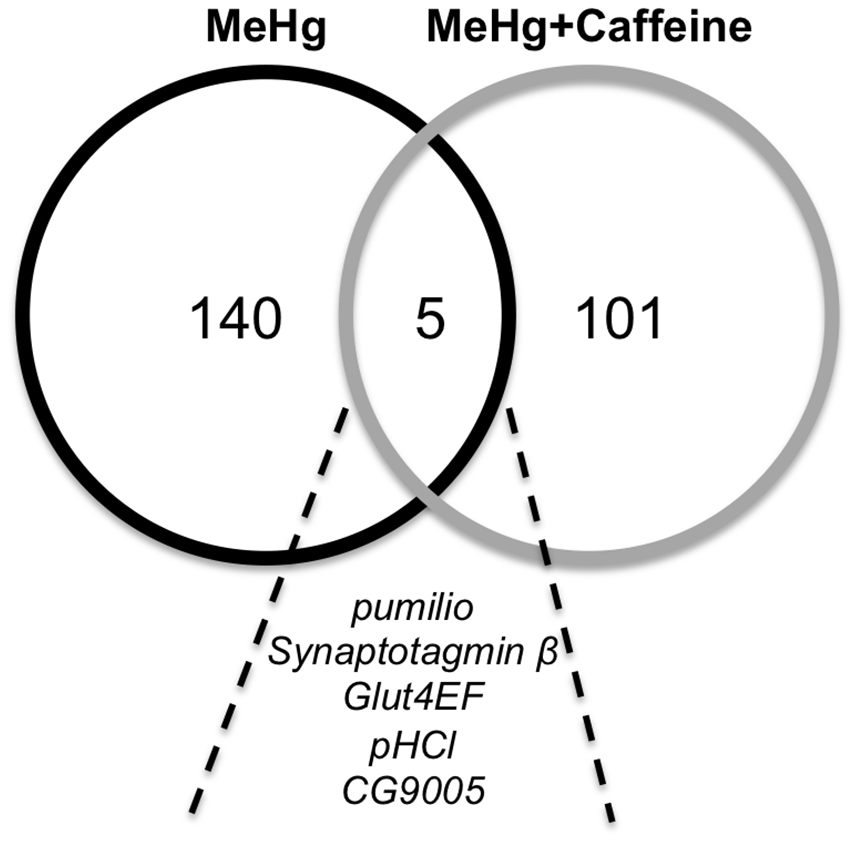
Overlap of genes identified by common polymorphic markers for variance in MeHg and MeHg+caffeine treatments. Candidate genes were identified from one or more associated polymorphisms in GWA analyses. A total of 145 and 106 genes were identified for MeHg and MeHg+caffeine, respectively. In common between the two treatments are 5 genes: *pumilio*, *Synaptotagmin* β, *Glut4EF*, *pHCl*, and *CG9005*.

We found five genes in common between the two GWA analyses for exposure to MeHg in the presence or absence of caffeine: *pumilio* (*pum*, *CG9755*), *Synaptotagmin β* (*Sytβ*, *CG42333*), *Glut4EF* (*CG34360*), *pHCl* (*CG44099*) and *CG9005* (Fig. 4). *pum* is an Armadillo repeat RNA binding protein involved in repression of translation and has two human homologs, PUM1 and PUM2. *pum* has functional implications in cellular and developmental processes including embryonic patterning, synaptic transmission, dendrite morphogenesis, pole cell migration, and learning and memory [28], [29]. *Sytβ* is one of seven Synaptotagmin family members in *Drosophila* with predicted function in synaptic vesicle exocytosis [30]. *Sytβ* localizes to the developing CNS as well as to specific motor neuron termini [31]. *Glut4EF* is a zinc finger transcription factor and homolog of the human glucose transporter (GLUT4) enhancer factor [32]. In flies, *Glut4EF* influences wing positioning with mutants giving a stretched out wing phenotype [32]. Mammalian Glut4EF is important for glucose uptake in muscles and associates with the MEF2A transcription factor [33]. *pHCl* encodes a gamma-aminobutyric acid A receptor, a ligand gated chloride channel that is expressed in the embryonic nervous system [34]. *CG9005* encodes a protein of unknown function that has two human homologs, Hsap/KIAA1370 and Hsap/FAM214B. RNAi knockdown of *CG9005* in flies has effects on development of the notum [35]. Therefore, four of the five overlapping genes share functions in the domain of neuromuscular function and neural development, suggesting that these biological processes are likely associated with MeHg tolerance during development.

A particularly noteworthy gene identified by GWA with MeHg alone is the metal transcription factor (*MTF-1*) gene, which was represented with six SNPs (Table S1). *MTF-1* is a conserved transcriptional regulator and the primary responder to heavy metal toxicity insult [36]. While better known for conferring resistance to divalent metal ions and inorganic mercury [37], *MTF-1* regulates expression of metallothionein proteins and is effective in moderating oxidative stress [38]. MTF-1 responds robustly to inorganic mercury in *Drosophila*; however, its function in MeHg tolerance in flies remains to be investigated.

### Functional gene networks related to MeHg tolerance

**Figure 5:**
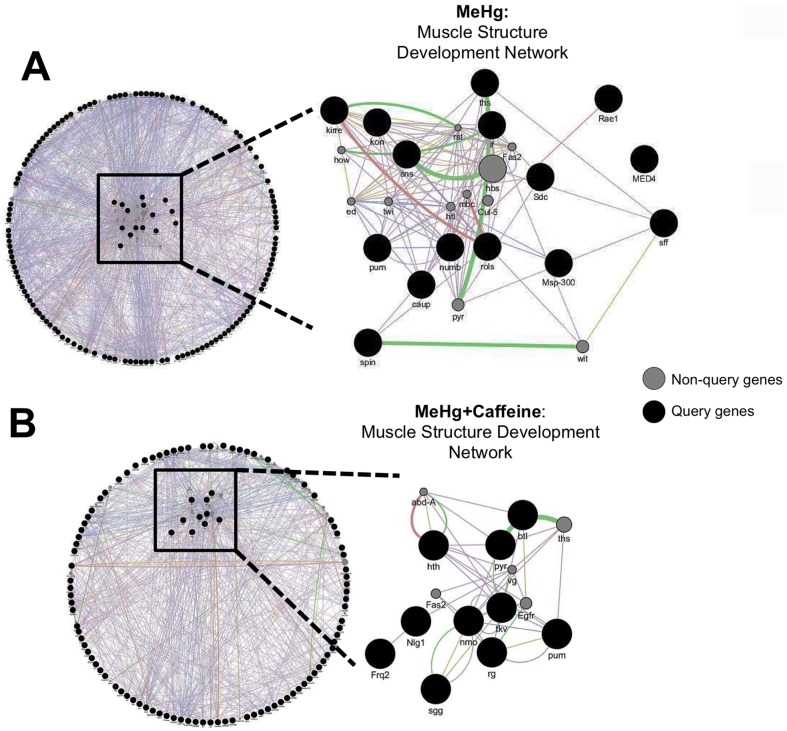
A shared functional network among non-overlapping genes identified in GWA of MeHg alone and MeHg+caffeine treatments. Network maps generated by GeneMANIA illustrate an example of a shared network for muscle structure development of genes identified in the GWA for MeHg alone (A) and MeHg+caffeine (B). Interactions are identified at the level of co-expression (purple lines), co-localization (blue lines), genetic interactions (green lines) and physical interactions (red lines). Query genes from the GWA analyses are represented by black circles and non-query genes (computationally recruited to complete network associations) are indicated by gray circles. The cluster of genes comprising the network is sorted to the inside of the circle, while the remaining query genes, not associated with the network, remain on the periphery.

To assess to what extent genes associated with variation in MeHg tolerance can be assembled into genetic networks that represent biological pathways, we used the GeneMANIA [39] algorithm. The results from such analyses generate hypotheses regarding biological processes associated with phenotypic variation that can subsequently be tested by disrupting hub genes though mutational analyses or targeted RNAi. Although only five candidate genes overlapped between the MeHg alone versus MeHg+caffeine treatments, we found substantial enrichment for genes associated with a number of muscle development functions for MeHg treatment both with and without caffeine and an additional overlap for the functional category of receptor protein kinase signaling (Table 3). One of the resulting GeneMANIA functional categories is assigned the “muscle structure development” network term and is shown schematically in Figure 5A and B for MeHg alone and MeHg+caffeine, respectively. Genes on the query list that are enriched for the “muscle structure development” category under analysis are listed in Tables 4 and 5. Five core genes in the network derived from the GWA analysis of the effects of MeHg alone act in muscle cell fusion and muscle attachment and development: *inflated* (*if*, *CG9623*), *kin of irre* (*kirre*, a.k.a. *dumbfounded* (*duf*) *CG3653*), *sticks and stones* (*sns, CG331441*), *kon-tiki* (*kon*, *CG10275*) and *rolling pebbles* (*rols*, *CG32096*) (Fig. 5A and Table 4). Also of note are five genes that play a role in neuromuscular junction development and integrity; *sugar free frosting* (*sff*, *CG6114*), *rae1* (*CG9862*), *pum* (CG9755), *spinster* (*spin*, *CG8428*) and *Syndecan* (*Sdc*, *CG10497)* (Fig. 5A and Table 4). Nine of these 15 genes have human homologs (Table 4). Among the 10 genes in the “muscle structure development” network with MeHg+caffeine treatments eight have human homologs (Table 5). It is of interest that eight of these 10 genes are transcriptionally responsive to caffeine (Table 5).

**Table 3:** GeneMANIA network analyses reveals both overlapping and unique biological functions among GWA genes from MeHg alone and MeHg+caffeine.

MeHg alone				MeHg + Caffeine			
Function	FDR	Network	Genome	Function	FDR	Network	Genome
**muscle organ development**	1.73E–11	24	247	***transmembrane receptor protein tyrosine kinase signaling***	3.40E–05	12	129
**muscle structure development**	1.73E–11	26	294	***enzyme linked receptor protein signaling pathway***	1.26E–04	13	187
**muscle cell differentiation**	3.38E–10	20	184	**muscle organ development**	3.04E–04	14	247
**striated muscle cell differentiation**	7.56E–09	18	170	**muscle structure development**	3.04E–04	15	294
**muscle tissue development**	7.58E–08	16	151	regulation of cell differentiation	9.79E–04	14	283
**striated muscle tissue development**	7.58E–08	16	150	tissue morphogenesis	1.05E–03	14	289
**skeletal muscle organ development**	7.58E–08	17	173	protein phosphorylation	1.53E–03	13	258
**visceral muscle development**	1.83E–07	7	14	epithelium development	3.80E–03	13	286
**skeletal muscle tissue development**	2.34E–07	15	141	adult behavior	3.80E–03	9	126
urogenital system development	1.00E–06	11	71	genital disc development	4.04E–03	6	44
renal system development	1.00E–06	11	71	response to organic substance	4.98E–03	12	261
cell migration	1.25E–06	17	217	embryonic morphogenesis	4.98E–03	11	213
cell motility	2.31E–06	17	227	regulation of nervous system development	4.98E–03	10	176
localization of cell	3.63E–06	17	235	**skeletal muscle organ development**	4.98E–03	10	173
**regulation of muscle tissue development**	4.45E–06	10	65	regulation of cell development	4.98E–03	11	217
**regulation of striated muscle tissue development**	4.45E–06	10	65	**neuromuscular junction development**	5.23E–03	8	106
**regulation of muscle organ development**	8.81E–06	10	70	**skeletal muscle fiber development**	5.64E–03	8	108
tube development	2.30E–05	13	149	morphogenesis of an epithelium	5.64E–03	12	269
tissue migration	3.42E–05	8	44	**muscle cell differentiation**	5.76E–03	10	184
***transmembrane receptor protein tyrosine kinase sig.***	3.51E–05	12	129	**muscle tissue development**	6.63E–03	9	151
***enzyme linked receptor protein signaling pathway***	4.00E–05	14	187	**striated muscle tissue development**	6.63E–03	9	150
cell-cell adhesion	7.98E–05	10	90	synapse organization	6.63E–03	9	149
**striated muscle cell development**	9.81E–05	12	144	hindgut morphogenesis	6.63E–03	6	55
renal filtration cell differentiation	9.81E–05	5	12	**muscle fiber development**	6.80E–03	8	116

GeneMANIA derived biological network integration results from MeHg and MeHg+caffeine GWA genes. Overlapping networks with muscle related function (bold text)and receptor signaling/tyrosine kinase function (italicized bold text) are indicated. Also shown are False discovery rate (FDR) and coverage indicated by the number of the genes in the ‘Network’ with a given function relative to all the genes in the ‘Genome’ identified with that function.

**Table 4:** MeHg: Muscle structure development network query genes.

Gene Name	Fly Base ID	No. SNPs	Gene Function	Protein Function	CG #	Human Homolog
***If***	FBgn0001250	11	Axon guidance, muscle attachment, myofibril assembly	integrin alpha chain	CG9623	-
***kirre***	FBgn0028369	1	Myoblast fusion	immunoglobulin	CG3653	Kirrel/NEPH1
***Sff***	FBgn0036544	1	NMJ development	Protein serine/threonine kinase	CG6114	-
***Rae1***	FBgn0034646	1	Negative regulation of synaptic growth at NMJ	WD40 repeat	CG9862	RAE1
***Rols***	FBgn0041096	1	Myoblast fusion	zinc finger, ankyrin repeat	CG32096	-
***Kon***	FBgn0032683	1	Muscle attachment, muscle organ development, neurogenesis	laminin G domain, concanavalin A-like lectin	CG10275	CSPG4
***pum***	FBgn0003165	1	Regulation of synaptic growth at NMJ, synaptic transmission, dendrite morphogenesis, pole cell migration	RNA binding protein	CG9755	PUM1/PUM2
***Msp-300***	FBgn0261836	1	Skeletal muscle development, flight, locomotion	actin-binding, actinin-type, spectrin repeat	CG42768	
***caup***	FBgn0015919	1	Muscle cell fate commitment	Tale/IRO homebox family	CG10605	IRX3
***spin***	FBgn0086676	1	Regulation of synaptic growth at NMJ, locomotion, nervous system remodeling, glial migration	unknown	CG8428	SPNS1/2/3
***ths***	FBgn0033652	1	Myoblast migration, heart development	FGF receptor binding, growth factor	CG12443	-
***sns***	FBgn0024189	1	Myoblast fusion, nephrocyte diaphragm assembly	immunoglobulin	CG33141	NPHS1
***Sdc***	FBgn0010415	1	Regulation of synaptic growth at NMJ, motor neuron axon guidance	heparin sulfate proteoglycan, cytoskeletal binding	CG10497	-
***numb***	FBgn0002973	3	Muscle cell fate specification, neurogenesis	Notch binding	CG3779	NUMB/NUMBL
***MED4***	FBgn0035754	1	Muscle organ development	RNA Pol II cofactor activity	CG8609	MED4

Genes identified in the GWA for MeHg that constitute the **“**Muscle Structure Development” network are listed. The human homologs listed were identified in Flybase.

**Table 5:** MeHg+caffeine: Muscle structure development network query genes.

Gene Name	Fly Base ID	No. SNPs	Gene Function	Protein Function	CG #	Human Homolog	Caff induced
***Hth***	FBgn0001235	3	Somatic muscle development, peripheral nervous system development, neuron differentiation	DNA binding protein	CG17117	MEIS1/2/3	low
***Nmo***	FBgn0011817	1	Positive regulation of synaptic growth at NMJ, eye and wing development	Protein kinase	CG7892	NLK	Y
***pum***	FBgn0003165	1	Regulation of synaptic growth at NMJ, synaptic transmission, dendrite morphogenesis, Pole cell migration	RNA binding protein	CG9755	PUM1/PUM2	Y
***Tkv***	FBgn0003716	1	Positive regulation of synaptic growth at NMJ, neuromuscular synaptic transmission, TGF-β signaling pathway	TGF beta receptor/Serine-threonine kinase	CG14026	BMPR1/ACVR1	Y
***Pyr***	FBgn0033649	1	Myoblast migration, cardioblast differentiation	FGF receptor binding, growth factor	CG13194	-	Y
***Sgg***	FBgn0003371	1	Negative regulation of synaptic growth at NMJ, circadian clock, epithelial cell morphogenesis	Serine threonine kinase	CG2621	GSK3A/B	Y
***Nlg1***	FBgn0051146	1	NMJ development, synaptic growth at NMJ	Carboxyesterase	CG31146	-	N
***Frq2***	FBgn0083228	1	NMJ development, synaptic transmission, regulation of neurotransmitter secretion	Ca++ binding guanylate cyclase activator	CG5744	KCNIP/NCALD	low
***btl***	FBgn0005592	1	Negative regulation of axon extension, tracheal outgrowth/open tracheal system	Tyrosine Kinase	CG32134	FGFR1/2/3/5, RET	N
***rg***	FBgn0266098	1	Neuromuscular junction development, mushroom body development, short-term memory, compound eye	Protein kinase binding	CG44835	LRBA, NBEA	low

Genes identified in the GWA for MeHg+caffeine that constitute the “Muscle Structure Development” network are listed. The human homologs listed were identified in Flybase. In addition, response to caffeine for each gene was adapted from the modENCODE treatment expression data in the Flybase record for each gene.

In summary, although the genes identified largely differ between the GWA analysis of effects of MeHg alone and MeHg+caffeine, the top GWA genes in both cases were functionally enriched for playing roles in muscle and neuromuscular junction development. This suggests that genes affecting muscle development contribute to the mechanisms of tolerance to MeHg toxicity during development.

### Functional assessment of sensitivity of muscle development to MeHg toxicity

**Figure 6:**
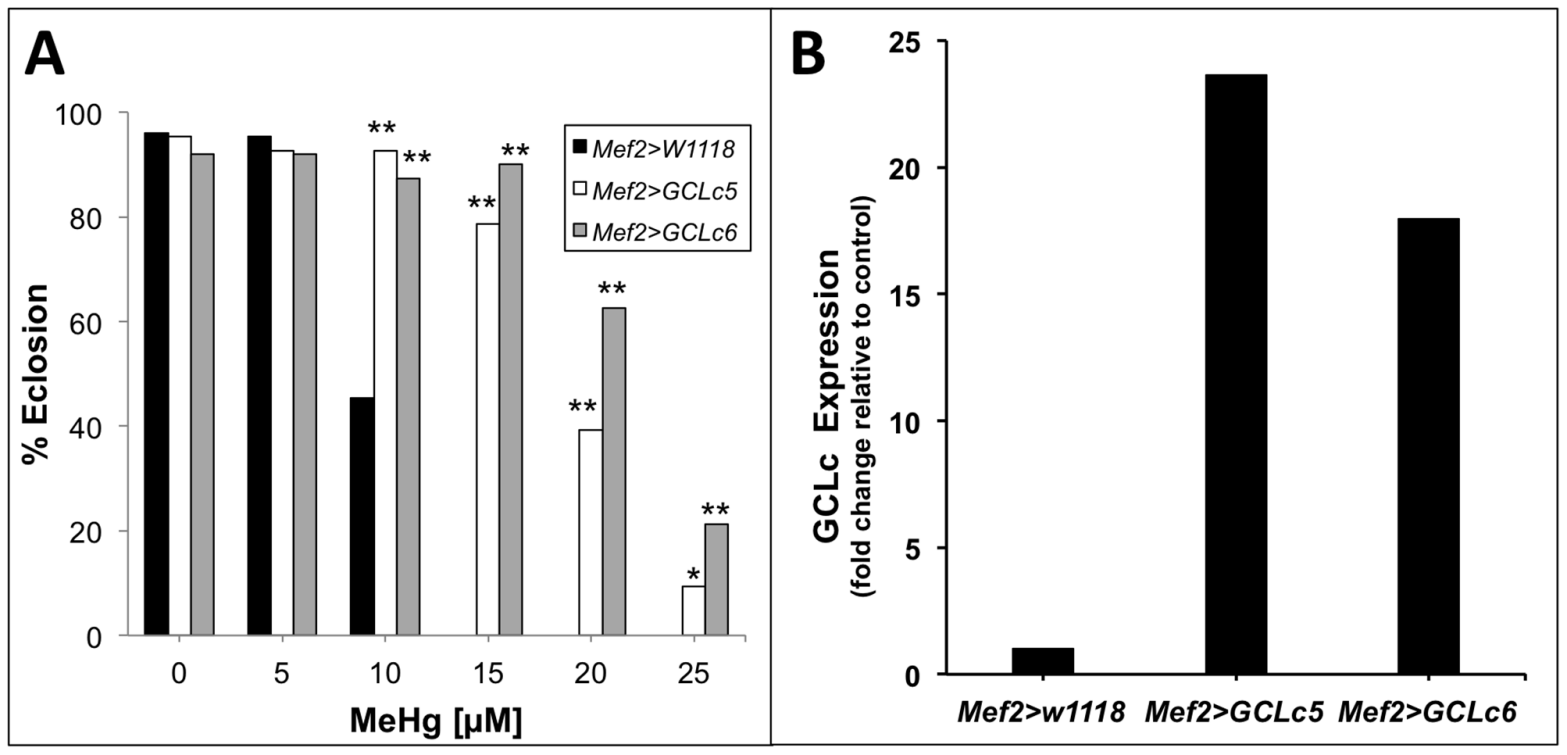
Muscle-specific expression of glutamate-cysteine ligase (GCL) rescues eclosion on MeHg. (A) Eclosion assays comparing flies expressing the catalytic subunit of GCL in two independent *UAS* responder lines (*GCLc5* and *GCLc6*) using the muscle-specific *Mef2-GAL4* driver (Mef2) with control flies (*Mef2>w^1118^*). Statistical analyses by *z-* test, *n* = 150 flies/bar. * *p* <0.001 and **p<0.0001 relative to *Mef2>w^1118^*. (B) Expression of *GCLc* mRNA in P12 pupae thoracic RNA extracts (pooled sample of n = 20 pupae) measured via qRT-PCR.

To functionally assay developing muscle tissue as a sensitive MeHg target, we examined flies that carry a gene known to affect MeHg tolerance expressed under the muscle specific enhancer *myocyte enhancing factor* 2 (*Mef2*) [40]. We induced expression of glutamate-cysteine ligase (GCL), the highly conserved, rate-limiting enzyme for the synthesis of glutathione (GSH). GSH is a small molecule thiol compound present in all cells and is a first line of defense to MeHg toxicity, forming a conjugate that enhances MeHg excretion [41]. Elevated expression of GCL gives resistance to MeHg toxicity [42]. Using the *Gal4*>*UAS* system, we over-expressed the catalytic subunit of GCL (GCLc) using two independent lines carrying the *UAS-GCLc* construct (*GCLc5*, *GCLc6*) with the *Mef2-Gal4* driver (Fig. 6B). GCLc expression in muscle shows a robust enhancement of tolerance to MeHg in the eclosion assay (Fig. 6A), consistent with the notion that protection of muscle development is critical to overall development of the fly and completion of eclosion under MeHg toxicity stress.

**Figure 7:**
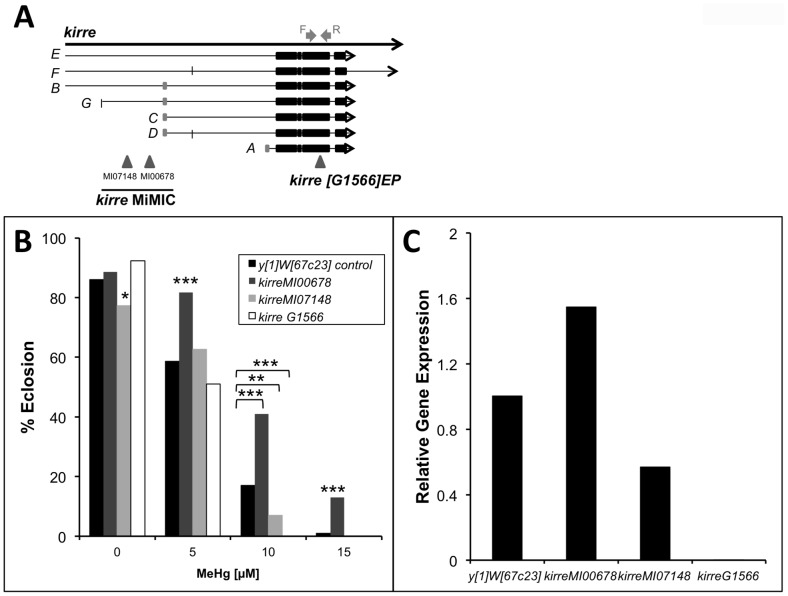
Altered MeHg tolerance in *kirre* mutant flies. (A) Schematic representation of mutant lines targeting *kirre*. *kirre^M107148^* is located at *X*: 2921499 targeting *kirre* transcripts E, F, B and G. *kirre* ^MI00678^ is located at *X*: 2952881 targeting E, F, B and G transcripts. *kirre* ^G1566^ is located at *X*: 3025507 in Exon 10 targeting all 7 splice variants of *kirre*. Gray arrows depict the location of the forward and reverse primers used for qRT-PCR analysis. *(*B) Eclosion assays of *kirre* mutant lines (MiMIC and EP) compared to *y^1^w^67c23^* control line. Statistical analyses done by z*-* test. *N* = 300 flies/bar. * *p*<0.01, **p<0.001 and *** *p*<0.0001 relative to *y^1^w^67c23^.* (C) Expression of *kirre* mRNA in P12 pupae thoracic RNA extracts (pooled sample of n = 20 pupae) measured via qRT-PCR.

We further examined flies carrying mutations in *kirre*, one of the core myogenic candidate genes identified in our GWA study. The *kirre^G1566^* mutation is a viable mutant of *kirre* carrying a *P*-element insert in exon 10 (Fig. 7A). The *kirre* ^MI07148^ and *kirre* ^MI00678^ mutations carry a *Minos* element insert in an intronic region of four of the seven transcripts (Fig. 7A). Analysis by qRT-PCR demonstrates that transcript levels are substantially reduced in the *kirre* ^MI07148^ mutant and essentially absent in the *kirre^G1566^* mutant (Fig. 7C), consistent with the predicted effect on transcripts encompassing the respective insertions. A moderate but significant reduction in MeHg tolerance is seen with both the *kirre* ^MI07148^ and *kirre^G1566^* mutants, specifically with exposure to 10 µM MeHg (Fig. 7B). Consistent with the corresponding expression levels, the *kirre^G1566^* mutant showed the lowest MeHg tolerance with the *kirre* ^MI07148^mutant giving an intermediate tolerance relative to the *yw* control line at 10 µM exposures (Fig. 7B). Unexpectedly, the *kirre* ^MI00678^ mutant shows a significant increase in MeHg tolerance at the 5, 10 and 15 µM exposures (Fig. 7B), and concomitantly, *kirre mRNA* levels are seen to be elevated relative to the *y* ^1^ *w* ^67c23^ control line (Fig. 7C). These findings are consistent with the notion that modulation in the levels of *kirre* expression affects sensitivity of muscle development to MeHg.

### Identification of an indirect flight muscle phenotype in MeHg-exposed pupae

**Figure 8:**
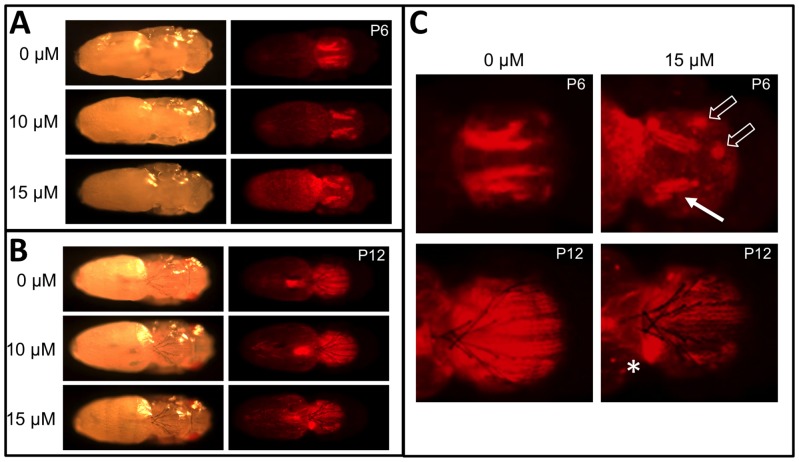
MeHg disruption of DLM muscle development. (A, B) *Mef2>RFP* pupae reared on indicated concentration of MeHg to stage P6 (A) or P12 (B) and imaged by bright light and red fluorescence to reveal DLM morphology. (C) Close-up image of selected panel from A and B. The solid arrow indicates reduced DLM bundle size and defects in DLM bundle splitting with MeHg. Open arrows indicate displacement of attachment sites of DVM bundles. Asterisks (*) indicates failure of extension and anchoring of the DLM resulting in myofibers coalesced in a ball. The development of eyes and bristles appear unaffected by MeHg treatment.

With MeHg exposure, eclosion commonly fails in late pupal stages resulting in the accumulation of dark pupae, particularly at 10–15 µM MeHg. Based on our GWA results we examined various stages of MeHg-exposed pupae for potential muscle phenotypes using a line of flies that expresses red fluorescent protein (RFP) constitutively in muscles (*Mef2*>RFP). *Mef2*>RFP larvae were exposed to various concentrations of MeHg and collected at various pupal stage endpoints. Fluorescent imaging of these pupae reveals the prominent pattern of the indirect flight muscle groups, notably the dorsal longitudinal muscles (DLMs) at their attachment sites under the notum. We observe an overall disruption of DLM morphogenesis with MeHg treatments, despite an apparent normal development of ectodermal structures as well as the specialized organs of the bristles and eyes (Fig. 8A, B). Following MeHg exposure, early pupae (P6) show a reduction in size of the DLM fiber bundles and an apparent displacement of forming DVM muscles (Fig. 8A, C upper panels, open arrows). At later stages (P12), in addition to reduced fiber size, a “ball” of RFP-positive tissue is seen indicative of a failure of DLM myofiber maturation, elongation and attachment to tendon cells on the notum epithelium (Fig. 8B, C lower panels).

## Discussion

### Natural variation in tolerance to MeHg toxicity

We sought to elaborate a fuller spectrum of the molecular networks and the tissue targets that influence MeHg toxicity outcomes through an unbiased query of polymorphisms across the entire genome of a diverse panel of developing animals. We find that variation in MeHg tolerance is under significant genetic control. Furthermore, we demonstrate that variation in response to a dietary modifier of MeHg toxicity, caffeine, is also genetically variable. This latter finding adds an additional level of complexity to interpreting outcomes of MeHg exposure, namely, that dietary factors may influence MeHg toxicity, but their efficacy, in itself, is subject to genetic pre-disposition. Nonetheless, for more than two thirds of the fly lines assayed, a beneficial effect of caffeine on MeHg toxicity was observed. Translating this finding to mammalian models and to humans has the potential to identify a means of moderating MeHg exposure effects through the diet.

### GWA identifies an association of MeHg tolerance with genes and networks in muscle development

We used a lenient statistical threshold of *p*<10^−4^ for identification of candidate genes associated with phenotypic variation. This threshold does not reach genome-wide significance based on Bonferroni multiple testing correction or permutation, but nonetheless serves as a hypothesis generating mechanism which identifies the top polymorphisms in a population where all polymorphisms are known. Candidate genes identified at this nominal threshold can be further verified through the use of mutants or by asking to what extent candidate genes are members of a network with a probability that is significantly higher than expected by chance.

The top candidate genes from our GWA analyses of MeHg tolerance were enriched for functions in muscle development. *kirre*(*duf*), *sns*, *if, kon* and *rols* are central players in myoblast fusion, myotube elongation and attachment and myofibrillogenesis in both embryonic and adult muscle development in flies [43]. Adult indirect flight muscles (IFMs) are an excellent model to study muscle development [43]. IFMs are comprised of dorsal longitudinal muscles (DLMs) and dorso-ventral muscles (DVMs), which act antagonistically. DLMs form through a process whereby persistent larval oblique muscles serve as templates for the recruitment and fusion of myoblasts that migrate from the notal region of the imaginal wing disc [44]. Myoblast homing and fusion to growing myotubes is mediated by *kirre* and *sns*, these being Ig-domain proteins and cognate ligand partners that mediate cell adhesion and the formation of multinucleate syncytial cells [45]. In this process, *kirre* interacts directly with *rols,* a scaffolding protein, to facilitate myoblast fusion [46]. Subsequent splitting of the growing myotube occurs to generate three and then six distinct fiber bundles in each hemithorax. Concurrently the bundles elongate with the tips eventually anchoring to tendon cell attachment sites, a process that requires *kon* [43] [47]. The fact that polymorphisms in these five core pathway genes are among the top associations with variation in MeHg tolerance strongly supports the hypothesis that muscle development is a MeHg target.

MeHg appears to disrupt myotube growth, fiber bundle splitting and, apparently, anchoring at the myotendinous junction in DLMs at the pupal stage (Fig. 8), a phenotype entirely consistent with disruption of the function of one or more genes listed above. Furthermore, *kirre* mutants that reduce expression levels demonstrated enhanced susceptibility to MeHg toxicity (Fig. 7). This effect was moderate and likely due to a redundant function for *kirre* and *roughest (rst*) [48]. Nonetheless, a *kirre* mutant causing increased expression results in a corresponding increase in MeHg tolerance (Fig. 7) reinforcing the notion that *kirre* can moderate MeHg toxic effects. A concerted role for muscle development in MeHg toxicity is strongly supported by our finding that pan-muscular expression of the MeHg protective enzyme, GCLc, gives a robust rescue of the MeHg effect on eclosion. How MeHg interacts with myogenic genes and/or their products remains to be characterized. A potential role of these genes is consistent with the notion that failure to eclose, a behavior that requires concerted contractions of newly formed adult muscles, results from MeHg disrupting the integrity of forming muscles.

Genes with a function in formation of the neuromuscular junction (NMJ) were also enriched in our network analyses, notably, *sff*, *pum*, *spin*, *Sdc*, *nmo*, *tkv*, *sgg*, *nlg1* and *frq2*. NMJ-related genes are more highly represented in the MeHg+caffeine treatment. Six of the seven genes with NMJ function have human homologs and, intriguingly, are induced by caffeine as indicted by modENCODE treatment data in the FlyBase entry for each gene. Overall, these findings support the notion that clinical motor deficits may stem from effects of MeHg at the level of the motor unit. NMJ establishment and maintenance relies upon coordinated expression of factors that mediate targeting of the growing axon and connecting nerve terminals with muscle fibers, which are subsequently reinforced through a mechanism reliant upon vesicular trafficking [49]. It is therefore plausible that MeHg tolerance could arise from a favorable expression profile of the above genes that is influenced by natural polymorphic variation and can be modulated by caffeine. The effects of MeHg on the electrophysiological function of mature NMJ is well characterized [50], however, the extent to which MeHg alters NMJ formation in development is not clear.

Our findings present a paradigm shift in the hypothesis that the developing nervous system is preferentially targeted and exceptionally sensitive to MeHg. Myogenesis and neurogenesis share several conserved molecular pathways, and furthermore, the development of motor systems is intrinsically reliant on coordinated signals between developing muscle cells and neurons. It is not surprising that myogenesis could be an equally sensitive target for MeHg and that neurological deficits in animal models and humans observed thus far could include a yet unappreciated neuromuscular component.

We have previously characterized a MeHg-specific activation of the Notch receptor target gene *Enhancer of split mDelta* (*E(spl)mδ)* in *Drosophila* cells and embryos [51]. *E(spl)mδ* is unexpectedly expressed in embryonic muscle precursors as well as in differentiated larval muscles at late embryonic stages [51]. Furthermore, MeHg exposure during embryo development, as well as ectopic *E(spl)mδ* expression in muscle precursors, results in disrupted muscle patterning and concomitant defects in motor neuron outgrowth and branching [51], [52]. While it remains to be seen if *E(spl)mδ* functions similarly in developing muscles at pupal stages, a recent study has identified a central role for Notch signals in maintaining migrating myoblast in a fusion incompetent state until encountering their myotube destination during IFM development [48]. Together, these data reinforce the notion that MeHg targets developing muscle in *Drosophila*, and further highlight the potential role for Notch signals, in addition to the gene candidates identified here, to mediate detrimental MeHg effects.

### Multiple functions of candidate genes in development and MeHg toxicity

Several of the genes identified here have pleiotropic functions in development. Importantly, a number of genes have central functions in neurogenesis, neuronal differentiation and axon outgrowth, for example, *numb, if, kon, spin and Sdc*. While several steps in muscle development rely on autonomous signaling mechanisms and muscle-specific cues, in certain contexts a neural scaffold is required for appropriate muscle development [53]. Notably, denervation of the individual IFM fibers during early pupal stages affect subsequent myoblast proliferation contributing to reduced muscle bundle size in the DLM and DVM [53]. Thus, the phenotypes seen here may reflect an underlying MeHg-sensitive neural mechanism that remains to be characterized.

Alternatively, natural variation in MeHg tolerance may stem from effects on development of organs critical for dealing with toxic insult and excretion. *kirre* and *sns* are fundamental for morphogenesis of the Garland cell nephrocyte, a major site of waste removal and filtration of insect hemolymph [45], [54]. *kirre/sns* function analogously to their vertebrate orthologs Neph1 and Nephrin, which direct morphogenesis of the slit diaphragm of the podocyte in the mammalian glomerulus of the kidney [55], [56]. In addition, *rols* functions in the normal morphogenesis of the Malpighian tubule, the renal organ of the fly [57]. Therefore, *kirre, sns* and *rols* may also influence MeHg tolerance by supporting development of essential excretory organs in addition to directing proper myogenesis during development.

### Caffeine as a modulator of MeHg toxicity

We found that caffeine modulates MeHg toxicity, and in most cases shows an enhancement of tolerance to MeHg. Interestingly, caffeine has a positive impact on neurodegenerative disease, such as Parkinson's disease [58]. However, in some cases caffeine shows a negative effect on development with MeHg. There is a strong genetic component to the natural variation of the MeHg modulating effect of caffeine in flies, which parallels reports of genetic variation in caffeine effects in humans [59]. These findings emphasize the need to approach the issue of MeHg tolerance and susceptibility with a greater understanding of the role of individual genetic background, as well as dietary behaviors, particularly in investigations of fish-eating human population studies where MeHg exposures are most common.

## Summary

We have identified muscle development as a prominent target for MeHg by associating genetic variation in the DGRP with MeHg toxicity. This finding expands the window of inquiry into mechanisms of MeHg toxicity. Candidate genes identified here set the stage for translational studies in vertebrates, and possibly in human populations, to assess to what extent muscle morphogenesis is compromised by this ubiquitous environmental toxin.

## Supporting Information

Figure S1 **Q-Q plots for eclosion train exhibited under MeHg alone and MeHg+caffeine treatments.**
(TIF)

Figure S2 **Q-Q plots for P-values under MeHg alone and MeHg+caffeine treatments.**
(TIF)

Table S1 **Genome Wide Association results for MeHg alone.** Minor and major allele identities and counts are indicated for each polymorphism (SNP =  single nucleotide polymorphism; INS =  insertion; DEL =  deletion; MNP = multiple nucleotide polymorphism). P-values and false discovery rates (FDR) are also indicated (See Materials and Methods). Gene identifications include the Flybase gene ID number (FB ID), gene name and location of the polymorphism.
(XLSX)

Table S2 **Genome Wide Association results for MeHg + Caffeine.** (See legend for Table S2.)
(XLSX)

Table S3 **Eclosion assay raw data.** Results of individual trials for eclosion assays are presented for each RAL line on food containing MeHg (concentration in µM) and Caffeine (2 mM) indicated in the column header. Results are expressed in percent (%) eclosion for 50 L1 larvae assayed in each trial (except for a few trials were 30 L1 larvae were assayed, indicated by italics).
(XLSX)
