# Supplementary figures and images for: Genome-Wide Association Analysis of Tolerance to Methylmercury Toxicity in Drosophila Implicates Myogenic and Neuromuscular Developmental Pathways
Source: PLoS One. 2014 Oct 31;9(10):e110375. doi: 10.1371/journal.pone.0110375 (PMC4215868; doi:10.1371/journal.pone.0110375)

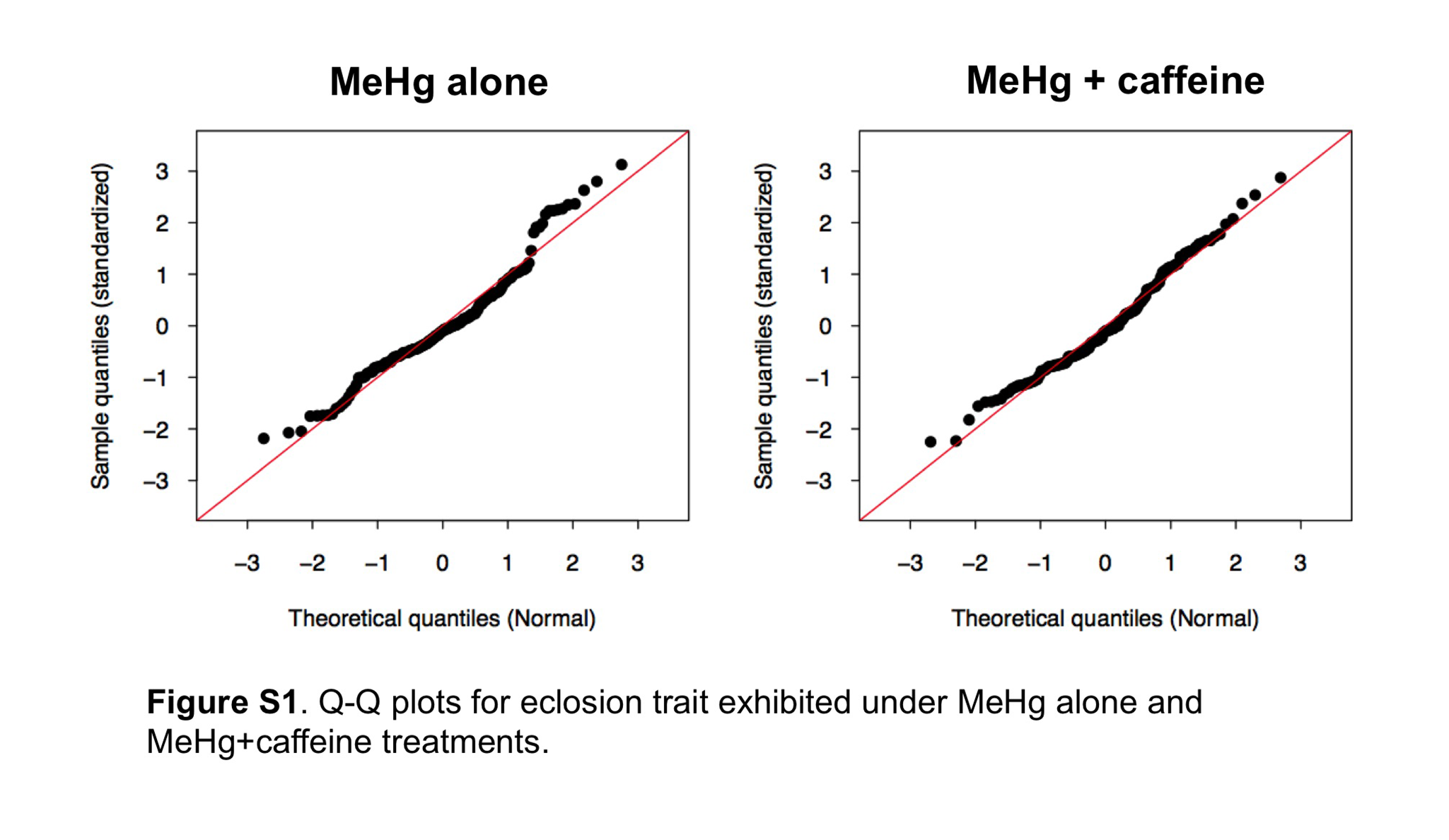

Supplement: Figure S1 — Q-Q plots for eclosion train exhibited under MeHg alone and MeHg+caffeine treatments. (TIF) [file pone.0110375.s001.tif]

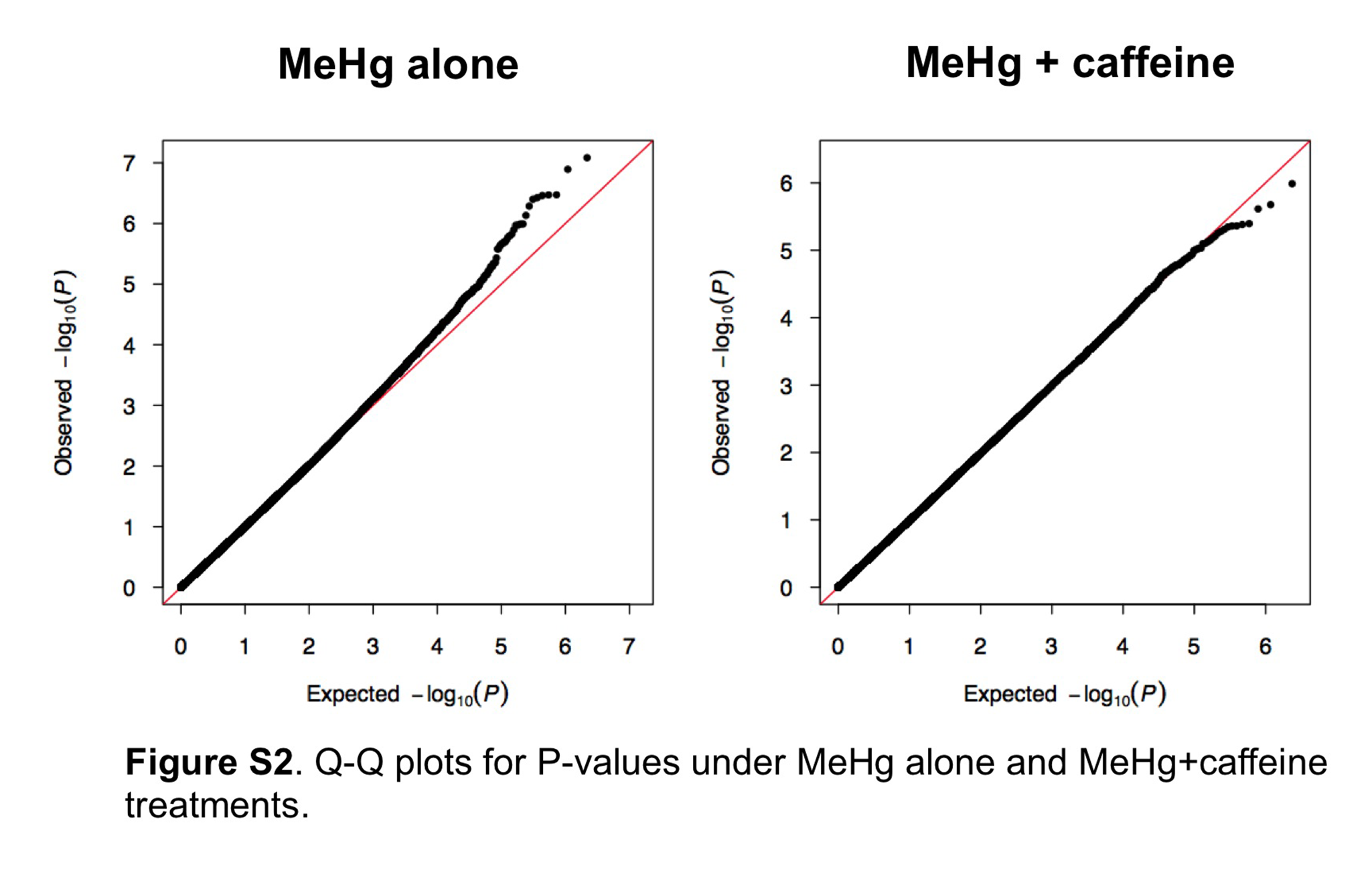

Supplement: Figure S2 — Q-Q plots for P-values under MeHg alone and MeHg+caffeine treatments. (TIF) [file pone.0110375.s002.tif]
